# Position paper on the safety/efficacy profile of Direct Oral Anticoagulants in patients with Chronic Kidney Disease: Consensus document of Società Italiana di Nefrologia (SIN), Federazione Centri per la diagnosi della trombosi e la Sorveglianza delle terapie Antitrombotiche (FCSA) and Società Italiana per lo Studio dell’Emostasi e della Trombosi (SISET)

**DOI:** 10.1007/s40620-020-00768-3

**Published:** 2020-07-31

**Authors:** Elvira Grandone, Filippo Aucella, Doris Barcellona, Giuliano Brunori, Giacomo Forneris, Paolo Gresele, Marco Marietta, Daniela Poli, Sophie Testa, Armando Tripodi, Simonetta Genovesi

**Affiliations:** 1 Thrombosis and Haemostasis Unit, Fondazione IRCCS “Casa Sollievo della Sofferenza”, San Giovanni Rotondo, Italy; 2 Ob/Gyn Department of the First I.M. Sechenov, Moscow State Medical University, Moscow, Russia; 3 Nephrology and Dialysis Unit, Fondazione IRCCS “Casa Sollievo della Sofferenza”, San Giovanni Rotondo, Italy; 4 Department of Medical Sciences and Public Health, University of Cagliari, Cagliari, Italy; 5 Nephrology and Dialysis Unit, S. Chiara Hospital, Trento, Italy; 6 Department of Clinical and Biological Sciences, University of Turin, Turin, Italy; 7 Department of Medicine, Section of Internal and Cardiovascular Medicine, University of Perugia, Perugia, Italy; 8 Department of Medical and Surgical Sciences, Section of Hematology, University of Modena and Reggio Emilia, Modena, Italy; 9 Center for Atherothrombotic Diseases, Azienda Ospedaliero-Universitaria Careggi, Firenze, Italy; 10 Haemostasis and Thrombosis Center, ASST Cremona, Cremona, Italy; 11 Angelo Bianchi Bonomi Hemophilia and Thrombosis Center and Fondazione Luigi Villa, Fondazione IRCCS Ca’ Granda Ospedale Maggiore Policlinico, Milano, Italy; 12 University of Milan-Bicocca, Milano, Italy; 13 Nephrology Unit, San Gerardo Hospital, Monza, Italy; 14 SHRO Temple University, Philadelphia, USA

**Keywords:** Direct oral anticoagulants, Glomerular filtration rate, Chronic kidney disease, Elderly, Polypharmacy

## Abstract

Direct oral anticoagulants (DOAC) are mostly prescribed to prevent cardioembolic stroke in patients with non-valvular atrial fibrillation (AF). An increasing number of guidelines recommend DOAC in AF patients with preserved renal function for the prevention of thromboembolism and an increased use of DOAC in daily practice is recorded also in elderly patients. Aging is associated with a reduction of glomerular filtration rate and impaired renal function, regardless of the cause, increases the risk of bleeding. Multiple medication use (polypharmacy) for treating superimposed co-morbidities is common in both elderly and chronic kidney disease (CKD) patients and drug-drug interaction may cause accumulation of DOAC, thereby increasing the risk of bleeding. There is uncertainty on the safety profile of DOAC in patients with CKD, particularly in those with severely impaired renal function or end stage renal disease, due to the heterogeneity of studies and the relative paucity of data. This document reports the position of three Italian scientific societies engaged in the management of patients with atrial fibrillation who are treated with DOAC and present with CKD.

## Introduction

Vitamin K antagonists (VKA) and direct oral anticoagulants (DOAC) are widely prescribed for non-valvular atrial fibrillation (AF) and treatment/prophylaxis of venous thromboembolism. There is a large body of literature in favor of DOAC for the prevention of cardioembolic stroke in patients with preserved renal function and an increasing number of guidelines now recommend DOAC for this and other clinical indications [1]. This has led to an increased use of DOAC in daily practice also in elderly and frail patients.

Polypharmacy is frequent in elderly individuals because of the presence of several co-morbidities and many important interactions between several drugs and VKA or DOAC have been reported. The interaction between VKA and drugs that modify cytochrome 2C9 and/or 3A4 is well known, as well as the interaction between DOAC and drugs that modify p-glycoprotein or cytochrome 3A4 [2].

Glomerular filtration rate (GFR) decreases progressively with age, thus increasing the risk of bleeding. DOAC undergo renal elimination to a variable extent (dabigatran about 80%, rivaroxaban 36%, apixaban 27% and edoxaban 50%) and can therefore accumulate in patients with declining renal function. Hence, dose adjustment is recommended [3]. Indeed, in young and healthy patients the pharmacokinetic profile of these medications is more predictable than in elderly multimorbid and polypharmacy patients. Except for dabigatran, which is removed by 50–60% with a single dialysis session, other DOAC are difficult to dialyze due to their high binding to plasma proteins [4].

When bleeding complications occur or in case of urgent/emergency surgery, the effect of dabigatran can be reversed using idarucizumab, whereas the activity of FXa inhibitors can be reversed by andexanet alfa, that is already on the market in the US and will be soon available in Europe. However, there is limited experience with these antidotes in clinical practice, as the number of patients so far treated is rather limited [5].

Very recently, hemoperfusion by means of an approved adsorption device has been shown to be able to remove rivaroxaban and dabigatran in vivo [6, 7]. These preliminary reports create the conditions for a possible all-inclusive method for elimination of dabigatran and FXa antagonists using a Cytosorb absorption column.

There is uncertainty on the safety profile of DOAC in patients with chronic kidney disease (CKD), particularly in those with severely impaired renal function or end stage renal disease (ESRD) [8]. On the other hand, in CKD patients AF is associated with an increased rate of ESRD [9, 10] and it has been described that oral anticoagulant treatment in these patients is associated with an increased risk of bleeding events [11]. Table 1 summarizes the present position of different scientific societies with regard to the use of DOAC in patients with CKD.

**Table 1:** Recommendations by scientific societies

Guideline	eGFR 30–50 ml/min (Cockroft-Gault formula)	eGFR 15–30 ml/min (Cockroft-Gault formula)	eGFR < 15 ml/min (Cockroft-Gault formula) or ESRD
ESC, Steffel (2018) [63]*	110 mg bid Dabigatran 15 mg/die Rivaroxaban 2.5 mg bid Apixaban if weight < 60 kg or age > 80 yrs; 30 mg/die Edoxaban	15 mg/die Rivaroxaban 2.5 mg bid Apixaban if weight < 60 kg or age > 80 yrs; 30 mg/die Edoxaban	Avoid DOAC
AHA, January (2019) [64]*	150–110 mg bid Dabigatran 15 mg/die Rivaroxaban 2.5 mg bid Apixaban if creatinine > 1.5 mg/dl and weight < 60 kg or age > 80 yrs; 30 mg/die Edoxaban	75 mg bid Dabigatran 15 mg/die Rivaroxaban 2.5 mg bid Apixaban if creatinine ≥ 1.5 mg/dl and weight < 60 kg or age > 80 yrs; 30 mg/die Edoxaban	2.5 or 5 mg bid Apixaban might be considered
KDIGO, Turakhia (2018) [53]	110 mg bid Dabigatran 15 mg/die Rivaroxaban 2.5 mg bid Apixaban 30 mg/die Edoxaban	15 mg/die Rivaroxaban 2.5 mg bid Apixaban if creatinine ≥ 1.5 mg/dl and weight < 60 kg or age > 80 yrs; 30 mg/die Edoxaban	2.5 mg bid Apixaban might be considered

*ESRD* end stage renal disease, *ESC* European Society of Cardiology, *AHA* American Heart Association, *KDIGO* kidney disease improving global outcomes

*It is recommended to carefully monitor the renal function at an interval depending on the individual degree of renal dysfunction

In this position paper the CKD stages are defined according to the KDIGO 2012 Clinical Practice Guideline for the Evaluation and Management of Chronic Kidney Disease [12].

Although the Food and Drug Administration (FDA) approved DOAC use for patients with CKD stage G5 [13], none of the randomized controlled trials (RCT) on DOAC included patients with estimated GFR (eGFR) < 25 ml/min. Moreover, extreme caution is recommended as the FDA indications are mainly based on pharmacokinetic data. FDA allows the prescription of rivaroxaban (15 mg/day) and apixaban (5 mg twice a day) in patients with ESRD or undergoing dialysis if the body weight is > 60 kg or age < 80 years, whereas the European Medicines Agency (EMA) contraindicates the use of any DOAC in patients with CKD stage G4–G5.

This paper aims to report the position of three Italian scientific societies that are engaged in the management of patients on DOAC who present with CKD.

## Use of DOAC in CKD: available evidence on safety/efficacy profile in RCTs

The four most frequently used DOAC (dabigatran, rivaroxaban, apixaban and edoxaban) have been compared to warfarin in several RCTs. In all trials, the presence of CKD stage G4–G5 was considered an exclusion criterion [for dabigatran, rivaroxaban and edoxaban and eGFR < 25 ml/min or serum creatinine > 2.5 mg/dL for apixaban]. In the presence of eGFR < 50 mL/min, the doses of DOAC were reduced: from 150 to 110 mg twice daily for dabigatran, from 20 to 15 mg once a day for rivaroxaban, from 60 to 30 mg once daily for edoxaban. The dose of apixaban was reduced to 2.5 mg twice daily in the presence of serum creatinine > 1.5 mg/dL, but only if associated with a body weight < 60 kg and/or age > 80 years.

Since renal function was not considered as a randomization criterion in any RCT, data from the CKD patients subgroup recruited should be considered as *post-hoc* analysis results. All *post-hoc* analyses have shown that the performance of DOAC, both in terms of safety and efficacy, was maintained also in patients with CKD stage G1 to G3b [14–17]. A similar safety result was obtained from a recent *post-hoc* analysis including 269 G4 patients from the ARISTOTLE trial, treated with apixaban [18]. A systemic meta-analysis carried out by Zou et al. [19] that included five RCTs involving 72,608 patients, indicated that the risk of stroke was lower for DOAC- than for warfarin-treated patients with CKD stage 1 to 3b. In the same CKD stages DOAC were also associated with fewer major bleedings. In addition, there was a lower risk of hemorrhagic stroke in patients taking DOAC compared to warfarin [20]. However, among patients treated with DOAC, a lower dosage was associated with a higher risk of stroke or systemic embolism [19]. It is noteworthy that these data cannot be extended to the ESRD population, as the only available RCT (the RENAL-AF trial) showed a similar rate of major and clinically relevant non-major bleedings with apixaban and warfarin in these patients. However, this study was terminated prematurely and its power was limited by the small sample size. [RENal hemodialysis patients ALlocated apixaban versus warfarin in Atrial Fibrillation—RENAL-AF (NCT02942407). Abstract 20963, American Heart Association Annual Scientific Sessions, Philadelphia, 2019].

A systemic review with meta-regression analysis carried out by Nielsen et al. [21] included five RCTs (warfarin vs. DOAC) on 72,845 patients. The analysis suggests that apixaban and edoxaban are associated with a better safety profile in patients with CKD stages G1 to G3b. Actually, patients taking apixaban had less major bleeding events compared to those taking dabigatran (both doses), rivaroxaban or edoxaban at a dose of 60 mg, but not to those taking 30 mg of edoxaban. However, the analysis did neither provide any sufficient information on different dosages of apixaban and rivaroxaban nor supply any conclusions regarding to which DOAC is the best choice in this particular setting.

A Cochrane review reported that DOAC could be more effective than warfarin (moderate certainty evidence) in reducing the incidence of stroke and systemic embolism (five studies, 12,545 patients: RR 0.81, 95% CI 0.65–1.00) and major bleeding events (five studies, 12,521 patients: RR 0.79, 95% CI 0.59–1.04; low certainty evidence). However, these data were obtained mainly in patients (*n* = 12,155) with G3b stage, as the group of G4 stage consisted of only 390 patients [22].

**Statement**: Data from RCTs show that DOAC have at least similar safety and efficacy profiles in patients with CKD stages G2 to G3b and in patients with normal renal function.

**Statement**: In patients with CKD G4 DOAC should be used with caution because of lack of strong supporting evidence from RCTs. At present there are not enough data available to recommend the use of DOAC in patients with CKD G5 or on long term dialysis.

## Use of DOAC in CKD: available evidence on safety and efficacy profile in real-life studies

The main available real-life studies performed in CKD patients taking DOAC are summarized in Table 2. Since real-life studies often show mostly aggregated data without distinguishing patients treated with VKA from those treated with DOAC, it is not possible to draw meaningful conclusions regarding the subgroups of patients taking DOAC. Furthermore, these studies are mainly retrospective and therefore of limited value. A paper by Kumar et al. reports on 641 patients treated with DOAC, but the analysis does not allow to identify features of the CDK subgroup [23]. A retrospective Italian study showed a reduction in bleeding and a better efficacy profile in patients taking rivaroxaban compared to warfarin. However, some caution should be used in generalizing these data, as the events rate described in this study is very different from that of other studies (25 stroke episodes of which 15 hemorrhagic in 100 patients taking warfarin vs. none in 247 taking rivaroxaban) [24].

**Table 2:** Description of real-life studies including chronic kidney disease patients

Author	Study design	Patients taking DOAC (*n*)	DOAC type	HR (95% CI) bleeding/stroke (DOAC vs. reference group)	Reference group	Limitations
Lee (2015) [29]	Retrospective G3, G4 patients	59	Rivaroxaban Dabigatran	0.18 (0.07–0.45)/0.78 (0.21–3.00)	VKA	Small sample size
Chan (2015) [34]	Retrospective G5 patients	525	Rivaroxaban Dabigatran	1.38 (1.03–1.83) (Rivaroxaban); 1.48 (1.21–1.81) (Dabigatran)/na	VKA	Data on stroke not available
Harel (2016) [25]	Population-based nested case–control G3, G4 patients	570	Rivaroxaban Dabigatran	1.22 (0.83–1.79) (Rivaroxaban); 1.15 (0.91–1.45) (Dabigatran)/na	VKA	Administrative database Data on stroke not available
Stanton (2017) [26]	Retrospective G4 patients	78	Apixaban	No difference in bleeding and stroke	VKA	Small sample size
Sarrat (2017) [33]	Retrospective G5 patients	40	Apixaban	No difference in bleeding/na	VKA	Small sample size Data on stroke not available
Becattini (2018) [32]	Prospective G2, G3, G4 patients	449	Dabigatran Rivaroxaban Apixaban	1.02 (1.01–1.04) every 1 ml/min decrease in eGFR/na	Not worsening renal function	Data on stroke not available
Siontis (2018) [35]	Retrospective G5 patients	2351	Apixaban	0.71 (0.56–0.91)/1.11 (0.82–1.50) (2.5 mg bid) 0.71 (0.53–0.95)/0.64 (0.42–0.97) (5 mg bid)	VKA	Medicare beneficiaries
Kumar (2018) [23]	Population-based retrospective G3–G4 patients	641	Rivaroxaban Apixaban Dabigatran Edoxaban (4 patients)	2.42 (1.44–4.05)/2.60 (2.00–3.38) (DOAC + VKA)	No anticoagulation	Pooled data with VKA
Schafer (2018) [31]	Retrospective G4–G5 patients	302	Apixaban	No difference in bleeding and stroke at 0 to 3 months 0.16 (0.05–0.50) at 6–12 months/no difference at 6–12 months	VKA	Administrative Database
Shin (2018) [28]	Retrospective, G3–G4 pts	1168	Dabigatran Rivaroxaban Apixaban	1.23 (1.02–1.48)/1.02 (0.76–1.37)	VKA	Administrative database
Bonnemeier (2019) [27]	Retrospective, CKD patients	6102	Rivaroxaban	0.66 (0.38–1.14)/0.72 (0.55–0.94)	VKA	Administrative database
Coleman (2019) [30]	Retrospective, G4–G5 patients	1896	Rivaroxaban	− 32% (1–53%)/0.67(0.30–1.50)	VKA	Administrative database
Makani (2020) [65]	Retrospective, G3–G4–G5 patients	10,794	Dabigatran Rivaroxaban Apixaban Edoxaban (11 patients)	G3 0.83 (0.74–0.94)/na G4-G5 0.69 (0.50–0.93)/na	VKA	Compliance to treatment

*HR* hazard ratio, *CI* confidence interval, *VKA* vitamin K antagonist

With regard to bleeding, data in G3–G4 CKD patients are not univocal. Indeed, some authors report no difference in the incidence of bleeding events between DOAC and VKA [25–27], some describe a higher number of bleeding events in patients taking DOAC [28], while others report lower incidence in patients taking DOAC [29, 30]. On the other hand, most studies agree that DOAC and VKA are equally effective in the prevention of thromboembolic risk in CKD patients [25, 26, 28–31].

For what regards the use of DOAC in ESRD patients undergoing dialysis, there are no conclusive data.

Recently, a study on 449 patients with a follow-up of about 18 months showed that variations of eGFR over time are inversely and independently related with the risk of bleeding: 1 mL/min absolute decrease in eGFR was associated with a 2% increase in the risk of both major and non-major bleeding [32]*.*

Retrospective matched-cohort studies comparing apixaban and warfarin, performed in small numbers of severe CKD and ESRD patients, showed non-significant differences in the occurrence of major bleeding between DOAC and VKA groups, with similar rates of ischemic stroke [26, 31, 33]. In hemodialysis patients, Chan et al. [34] demonstrated a higher risk of death from bleeding with dabigatran and rivaroxaban compared to warfarin, whereas Siontis et al. [35] showed no difference in the rate of thromboembolic events between apixaban and warfarin, with a lower risk of bleeding in patients taking apixaban. Finally, a recent meta-analysis of observational studies carried out in patients on long-term dialysis showed that VKA, dabigatran and rivaroxaban are associated with a significantly higher bleeding risk compared with apixaban and no anticoagulant treatment. However, only two of the sixteen studies included had investigated DOAC and there was significant heterogeneity in the analysis. Consequently, the authors state that to draw conclusions on the benefit-to-risk ratio in patients on long-term dialysis RCTs are warranted [36].

There is some concern about the association between VKA and vascular calcifications [37], observed particularly in the elderly taking warfarin [38], even if some authors find that CKD patients are exposed to calcifications independently of warfarin intake [39, 40]. Indeed, vascular calcifications could expose to an increased risk of cardiovascular events [39–42]. In this respect, it has been suggested that DOAC may have a better performance in CKD patients [43]. One RCT performed in a population of hemodialysis patients with AF (VKA replacement by rivaroxaban with or without high dose of vitamin K) however, showed that withdrawal of VKA and addition of high-dose vitamin K2 improved the vitamin K status, but did not reduce the progression of vascular calcifications [44].

Warfarin-related nephropathy is a clinical-pathologic entity that is often underdiagnosed, which occurs in the setting of an INR of > 3.0 [42, 45] and leads to acute kidney injury. In addition, in CKD patients, AF is associated with a faster progression towards ESRD [9, 10].

It has been hypothesized that in AF patients the use of DOAC is associated with both a lower risk of acute kidney injury and a slowdown in the deterioration of renal function when compared to VKA. Even if the data that are available so far are promising, they are not conclusive however, due to the retrospective nature of these studies [10, 28, 30]. A recent meta-analysis including 189,483 patients from eleven RCTs and three observational database studies (119,188 patients on DOACs and 70,295 patients on VKA or acetylsalicylic acid) suggested a significantly lower risk of renal impairment in AF patients with DOAC versus VKAs/acetylsalicylic acid (HR 0.67, 95% CI 0.62–0.73). The results did not change when considering a stricter definition of renal impairment (HR 0.65, 95% CI 0.60–0.71). Final conclusions are not robust however, because the analysis was performed by pooling data deriving both from patients taking acetylsalicylic acid and from those on warfarin treatment [46].

**Statement**: Data from observational studies suggest that DOAC have at least similar efficacy and safety profiles as VKA in patients with CKD stage G2 to G3b.

**Statement**: In patients with CKD G4–G5, also on long-term dialysis, DOAC should be used with caution, because of the small sample size of patients investigated and heterogeneity of studies.

**Statement**: Data on the interaction between DOAC, vascular calcifications and worsening of renal function in CKD patients are still not conclusive.

## Dosing DOAC in CKD patients

Since 2013 the Subcommittee on control of anticoagulation of the scientific and standardization committee of the International Society on Thrombosis and Haemostasis (ISTH) has recommended to measure DOAC plasma levels in some special clinical circumstances including (1) active bleeding; (2) before surgery/invasive procedure when the patient has taken the drug in the previous 24 h, or longer if CKD stage 3b or higher; (3) patients with deteriorating renal function; (4) perioperative management; (5) identification of subtherapeutic or supratherapeutic levels in patients at the extremes of body weight [47].

The plasma concentration of DOAC can be measured by relatively simple functional assays for both FIIa (dabigatran) and FXa inhibitors (rivaroxaban, apixaban, edoxaban) [48]. The tests for dabigatran are the dilute thrombin time or the ecarin clotting (or chromogenic) assays, whereas anti-FXa drugs can be measured by the anti-FXa assay [49].

Several reports have shown that there is a great variability among patients on DOAC, and that plasma levels poorly correlate with eGFR [50, 51]. Furthermore, recent observations show that bleeding complications are more frequent in patients with higher DOAC plasma levels at peak [52].

Several scientific societies suggest to measure DOAC prior to invasive procedures or surgery independently of eGFR. This is particularly recommended in elderly patients, in whom eGFR can vary rapidly over time [49, 53]. However, accurate definition of reliable cut off values is still lacking and we are waiting for findings from ad hoc studies [54].

A prospective study carried out in 422 patients on DOAC has shown that interrupting DOAC administration 3 days before a procedure was associated with a minimal pre-procedural anticoagulant effect and that both the presence of CKD stage 3b or higher and treatment with antiarrhythmic drugs should require a longer period of DOAC discontinuation [55].

A relevant issue is the choice of the most accurate method to calculate eGFR. The reported RCTs used the Cockcroft-Gault equation, that is considered less accurate than the Modification of Diet in Renal Disease Study (MDRD) and the Chronic Kidney Disease Epidemiology Collaboration (CKD-EPI) equations [56–58]. In general, CKD-EPI and MDRD give the best estimation of GFR [59]. Moreover, from a practical point of view, as MDRD or CKD-EPI do not consider body weight, these equations are more suitable in daily practice in the clinical wards, especially for bed-ridden patients for whom an estimation of weight is difficult to obtain. However, it has been shown that either equations may overestimate eGFR in elderly patients [60–62].

AF in CKD patients confers a high risk of worsening renal function and progression to ESRD [9, 10]. On the one hand the decrease in eGFR is associated with a higher risk of major and non-major bleeding [31], on the other hand it is associated with a higher risk of drug overdosing. For these reasons it is recommended to carefully monitor renal function in patients taking DOAC.

**Statement**: Measuring plasma DOAC concentration is recommended in case of (1) active bleeding/thrombosis; (2) urgent/emergency surgery (3) acute states (inflammation, sepsis, dehydration and acute kidney injury stage 2–3) [12].

**Statement**: Every hospital should have laboratory tests at their disposal for measuring DOAC plasma levels.

**Statement**: Careful monitoring of renal function in patients taking DOAC is recommended to avoid the risk of overdosing.

### Definitions

Chronic kidney disease classification according to estimated glomerular filtration rate.

G1: > 90 ml/min.

G2: 60–89 ml/min.

G3: 30–59 ml/min- G3a: 45–49 ml/min- G3b: 30–44 ml/min.

G4: 15–29 ml/min.

G5: < 15 ml/min.
